# Development and external validation of nomograms to predict the risk of skeletal metastasis at the time of diagnosis and skeletal metastasis-free survival in nasopharyngeal carcinoma

**DOI:** 10.1186/s12885-017-3630-9

**Published:** 2017-09-06

**Authors:** Lin Yang, Liangping Xia, Yan Wang, Shasha He, Haiyang Chen, Shaobo Liang, Peijian Peng, Shaodong Hong, Yong Chen

**Affiliations:** 1 Sun Yat-sen University Cancer Center, 651 East Dong Feng Road, Guangzhou, 510060 China; 2 State Key Laboratory of Oncology in Southern China, Guangzhou, China; 3 Collaborative Innovation Center for Cancer Medicine, Guangzhou, China; 4 The Six Affiliated Hospital of Sun Yat-sen University, Guangzhou, China; 5 The First Hospital of Foshan, Foshan, China; 6 The Fifth Affiliated Hospital of Sun Yat-sen University, Zhuhai, China

**Keywords:** Nasopharyngeal carcinoma, Skeletal metastasis at the time of diagnosis (SMAD), Skeletal metastasis free survival (SMFS), Prognosis, Nomograms

## Abstract

**Background:**

The skeletal system is the most common site of distant metastasis in nasopharyngeal carcinoma (NPC); various prognostic factors have been reported for skeletal metastasis, though most studies have focused on a single factor. We aimed to establish nomograms to effectively predict skeletal metastasis at initial diagnosis (SMAD) and skeletal metastasis-free survival (SMFS) in NPC.

**Methods:**

A total of 2685 patients with NPC who received bone scintigraphy (BS) and/or 18F–deoxyglucose positron emission tomography/computed tomography (18F–FDG PET/CT) and 2496 patients without skeletal metastasis were retrospectively assessed to develop individual nomograms for SMAD and SMFS. The models were validated externally using separate cohorts of 1329 and 1231 patients treated at two other institutions.

**Results:**

Five independent prognostic factors were included in each nomogram. The SMAD nomogram had a significantly higher c-index than the TNM staging system (training cohort, *P* = 0.005; validation cohort, *P* < 0.001). The SMFS nomogram had significantly higher c-index values in the training and validation sets than the TNM staging system (*P* < 0.001 and *P* = 0.005, respectively). Three proposed risk stratification groups were created using the nomograms, and enabled significant discrimination of SMFS for each risk group.

**Conclusion:**

The prognostic nomograms established in this study enable accurate stratification of distinct risk groups for skeletal metastasis, which may improve counseling and facilitate individualized management of patients with NPC.

**Electronic supplementary material:**

The online version of this article (10.1186/s12885-017-3630-9) contains supplementary material, which is available to authorized users.

## Background

Nasopharyngeal carcinoma (NPC) is a malignant head and neck cancer with a distinct ethnic and geographic pattern of distribution; the highest incidences of NPC (30–80 cases per 10,000/year) are observed in southern China and South East Asia [1]. Developments in advanced imaging modalities and instrumentation have enabled more precise tumor staging. Currently, approximately 5–8% of cases of NPC have distant metastasis (M1) at first diagnosis; the skeleton is the most common distant metastasis site, representing 70% to 80% cases of M1 disease [2–4]. Distant metastasis at diagnosis is associated with poorer survival outcomes and reduced quality of life. Moreover, research on M1 disease is sparse due to the poor survival outcomes of patients with skeletal metastases. However, increasing evidence indicates long-term survival and even a complete response can be achieved among a small proportion of patients with skeletal metastases, especially those who receive aggressive treatment [5]. This indicates different treatment methods could significantly improve the prognosis of selected high-risk M1 cases. However, solely relying on the TNM classification to predict the outcomes of patients with skeletal metastasis may result in inaccurate assessment, leading to unnecessary treatment and financial burdens or – even worse – the patient receiving a suboptimal treatment strategy. Moreover, individualized follow-up and treatment strategies may be required for specific subgroups of patients with different risks of skeletal metastasis.

Bone scintigraphy (BS) remains is the leading diagnostic method for bone metastasis during initial work-up as it is widely available and low cost. However, BS is not routinely conducted during follow-up as it has a low diagnostic sensitivity, especially for early bone metastatic lesions; metastases mainly located in the bone marrow are frequently not detected by BS [6]. Although 18F–FDG PET/CT has a higher sensitivity than BS for detecting bone metastases in primary NPC, 18F–FDG PET/CT technique is expensive [7]. However, differentiation of malignant and benign lesions on BS and 18F–FDG PET remains problematic, even for experienced nuclear physicians.

As far as we are aware, research on the frequency of bone metastases at initial diagnosis (SMAD) and skeletal metastasis-free survival (SMFS) in NPC is rare and narrowly-focused [8–11]. The lack of such data hampers accurate patient staging and risk stratification and delays the design of more reliable treatment protocols, as the M1 category is a “catch-all” classification that includes patients whose treatment response could be potentially curable or incurable. Identifying subgroups of patients with different risks of bone metastasis could help determine the appropriate imaging techniques and follow-up timing in a more personalized manner. Furthermore, more accurate prediction of the risk of skeletal metastasis could provide valuable decision-making information for clinicians and patients.

Nomograms incorporate a variety of important factors and have been demonstrated to be reliable prediction tools for quantifying individual risk in cancer. Nomograms can provide more precise prognoses than the traditional TNM staging system in several tumor types. To date, there has been no attempt to establish nomograms to predict SMAD and SMFS in NPC. We hypothesized nomograms combining T category, N category and other objective laboratory indexes could generate more accurate predictive models for SMAD and SMFS. Therefore, we assessed the prognostic risk factors for SMAD and SMFS in a large cohort of patients with NPC and validated the resulting nomograms using an external cohort treated at two other institutions.

## Methods

### Training cohort

The training cohort was derived from patients treated at Sun Yat-sen University Cancer Center between and December, 2012. The inclusion criteria were: (i) pathologically confirmed NPC; (ii) complete pretreatment clinical information and laboratory data; (iii) BS and/or 18F–FDG PET/CT at diagnosis of NPC; and (iv) complete follow-up data. Exclusion criteria were incomplete follow-up data, death due to non-NPC-associated accident, or previous/synchronous malignant tumors. Ethical approval was obtained from the institutional review boards. The requirement for informed consent was waived as this was a retrospective study. The study protocol complied with the Declaration of Helsinki and was approved by the Ethics Committee of Sun Yat-sen University Cancer Center.

A standardized form was designed to retrieve all relevant data, including sociodemographic data (age, gender, smoking history, alcohol exposure, family history of malignant tumors, family history of NPC); baseline laboratory data including plasma Epstein-Barr virus (EBV) DNA copy number, serum calcium, serum magnesium, serum phosphorus, serum albumin(ALB), serum globulin (GLB), serum aspartate transaminase (AST), serum alanine transaminase (ALT), serum alkaline phosphatase (ALP), serum lactate dehydrogenase (LDH), serum C-reactive protein (CRP); T category [primary tumor location, size, extension], N category [number/location of lymph node metastases); and treatment data (radiotherapy technique, fractions, dosage; chemotherapy). Clinical stage was assessed using the seventh edition of the AJCC/UICC TNM staging system.

### Treatment

All patients were treated using definitive radiotherapy (RT). The dose ranges for the nasopharynx, node-positive region and node-negative regions were 60–80, 60–70, and 50–60 Gy, respectively. Patients with stage I or II NPC did not receive chemotherapy; patients with stage III or IV NPC received induction, concurrent or adjuvant chemotherapy (or a combination of these strategies) as recommended by the institutional guidelines. Induction or adjuvant chemotherapy were cisplatin with 5-fluorouracil; cisplatin with taxoids; or cisplatin, 5-fluorouracil and taxoids (every 3 weeks; two to three cycles). Concurrent chemotherapy was cisplatin in weeks 1, 4 and 7 of radiotherapy or cisplatin weekly.

### Validation cohort

To examine the general applicability of the model, an independent external validation cohort of 1329 consecutive patients with NPC who received definitive radiotherapy at the Fifth affiliated hospital of Sun-Yat Sen University and the First hospital of the Foshan between January, 2006 and December, 2012 were included. Inclusion and exclusion were the same as the training cohort. Sufficient data was available for all patients to score all variables in the nomograms established in this study.

### Statistical analysis

SMAD was defined as the presence of skeletal metastasis on BS or 18F–FDG PET/CT at initial diagnosis (before receiving any treatment). SMFS was measured as time from diagnosis to detection of skeletal metastasis or censorship at last follow-up. In the training set, continuous variables were expressed as mean (± standard deviation), medians and ranges were transformed into dichotomous variables using the median value. Categorical variables were compared using the chi-square test or Fisher’s exact test; categorical/continuous variables, univariate logistic regression. Variables achieving significance at the level of *P* < 0.05 were entered into multivariate logistic regression analyses via stepwise procedures. In the training set, survival curves for different variables were plotted using the Kaplan-Meier method and compared using the log-rank test. Significant variables (*P* < 0.05) were entered into the Cox proportional hazards multivariate analyses to identify independent prognostic factors via forward stepwise procedures (*P* < 0.05). Statistical data analyses were performed using SPSS 22.0 (SPSS, Chicago, IL, USA).

Based on multivariate analyses, nomograms were generated to provide visualized risk prediction using the survival and rms packages of R 2.14.1 (http://www.r-project.org). Nomograms were subjected to bootstrap resampling (*n* = 1000) for interval and external validation to correct the concordance index (c-index) and explain variance with respect to over-optimism. The ability of the nomograms and TNM staging system to predict survival were compared using the c-index, a variable equivalent to the area under curve (AUC) of receiver operating characteristic curves for censored data. The maximum c-index value is 1.0, which indicates perfect prediction, while 0.5 indicates the probability of correctly predicting the outcomes by random chance. The nomogram and TNM staging system were compared using rcorrp.cens in the Hmisc module of R. The nomogram for 1-, 3-, and 5-year SMFS was calibrated by comparing predicted and actual observed survival rates. During external validation, the nomogram point scores were calculated for individual patients, then Cox regression analysis was performed using total point scores as a predictor in the validation cohort.

In addition to numerically comparing discriminative ability by c-index, we also attempted to confirm the superior independent discriminative ability of the nomograms over the standard TNM staging system. The training cohort were evenly grouped into three risk groups by nomogram score, then we investigated the predictive ability of the risk stratification cut-off points and different subgroups (TNM stage) using Kaplan-Meier survival curve analysis. A two-sided *P* value <0.05 was deemed significant. Details of the R code used to generate the nomograms can be assessed in the additional information online (Additional file 1). This trial was registered with Clinical Trials.Gov (NCT00705627); all data has been deposited at Sun Yat-sen University Cancer Center for future reference (number RDD RDDA2017000293).

## Results

### Patient characteristics and survival

A total of 2685 and 1329 patients in the training and external validation cohorts were eligible for the SMAD analyses (Additional file 2: Figure S1). Median age was 45-years-old (range, 23 to 78-years-old) for the training cohort and 45-years-old (range, 19 to 70-years-old) for the validation cohort. After excluding patients with distant metastasis at diagnosis, 2469 and 1231 patients were included in the analyses for SMFS. Median follow-up for SMFS in the training cohort was 65.0 months and 61.8 months in the validation cohort. Five-year SMFS was 86% in the training cohort and 85.4.0% in the validation cohort. In both cohorts, a total of 391 patients (9.7%) developed skeletal metastases after initial diagnosis, and 287 patients (7.7%) were confirmed to have skeletal metastases at initial diagnosis. The characteristics of the cohorts are summarized in Table 1 and Additional file 3: Table S1.

**Table 1:** Associations between the clinical and laboratory characteristics of the patients and SMAD as indicated by the chi-square test or Fisher’s exact test

Characteristic	Number (%)	Training cohort		*P*-value	Validation cohort Number (%)
		SMAD			
		Absent	Present		
Age, years				0.379	
< 45	1404 (52.3%)	1311 (93.4%)	93 (6.6%)		679 (51.1%)
≥ 45	1281 (47.7%)	1185 (92.5%)	96 (7.5%)		650 (48.9%)
Sex				0.025	
Male	2131 (79.4%)	1969 (92.4%)	162 (7.6%)		986 (74.2%)
Female	554 (20.6%)	527 (95.1%)	27 (4.9%)		343 (25.8%)
Smoking Status				0.055	
Absent	1708 (63.3%)	1600 (93.7%)	108 (6.3%)		795 (59.8%)
Present	977 (36.4%)	896 (91.7%)	81 (8.3%)		534 (40.2%)
Drinking Status				0.873	
Absent	2382 (88.7%)	2215 (93.0%)	167 (7.0%)		1117 (84.0%)
Present	303 (11.3%)	281 (92.7%)	22 (7.3%)		212 (16.0%)
Family history				0.566	
Absent	1926 (71.7%)	1787 (92.8%)	139 (7.2%)		967 (72.8%)
Present	759 (28.3%)	709 (93.4%)	50 (6.6%)		362 (27.2%)
Calcium, mmol/L				0.932	
< 2.4	1370 (51.0%)	1273 (92.9%)	97 (7.1%)		501 (37.7%)
≥ 2.4	1315 (49.0%)	1223 (93.0%)	92 (7.0%)		828 (62.3%)
Phosphorus, mmol/L				0.587	
< 1.15	1398 (52.1%)	1296 (92.7%)	102 (7.3%)		676 (50.9%)
≥ 1.15	1287 (47.9%)	1200 (93.2%)	87 (6.8%)		653 (49.1%)
Magnesium, mmol/L				0.308	
< 0.93	1410 (52.2%)	1304 (92.5%)	106 (7.5%)		919 (69.1%)
≥ 0.93	1275 (47.5%)	1192 (93.5%)	83 (6.5%)		410 (30.9%)
CRP, mg/L				< 0.001	
< 1.91	1345 (50.1%)	1283 (95.4%)	62 (4.6%)		722 (54.3%)
≥ 1.91	1340 (49.9%)	1213 (90.5%)	127 (9.5%)		607 (45.7%)
WBCs, ×10^9^				0.137	
< 6.9	1376 (51.2%)	1289 (93.7%)	87 (6.3%)		677 (50.9%)
≥ 6.9	1309 (48.8%)	1207 (92.2%)	102 (7.8%)		652 (49.1%)
Neutrophils, ×10^9^				0.001	
< 4.2	1356 (50.5%)	1283 (94.6%)	73 (5.4%)		691 (52.0%)
≥ 4.2	1329 (49.5%)	1213 (91.3%)	116 (8.7%)		638 (48.0%)
HGB, g/L					
< 145	1379 (51.4%)	1264 (91.7%)	115 (8.3%)	0.007	758 (57.0%)
≥ 145	1306 (48.6%)	1232 (94.3%)	74 (5.7%)		571 (43.0%)
Platelets, ×10^9^				0.013	
< 229	1343 (50.0%)	1265 (94.2%)	78 (5.8%)		638 (48.0%)
≥ 229	1342 (50.0%)	1231 (91.7%)	111 (8.3%)		691 (52.0%)
ALT, U/L				0.392	
< 22.2	1345 (50.1%)	1256 (93.4%)	89 (6.6%)		725 (54.6%)
≥ 22.2	1340 (49.9%)	1240 (92.5%)	100 (7.5%)		604 (45.4%)
AST, U/L				0.092	
< 21	1366 (50.9)	1281 (93.8%)	85 (6.2%)		675 (50.8%)
≥ 21	1319 (49.1%)	1215 (92.1%)	104 (7.9%)		654 (49.2%)
ALP, U/L				< 0.001	
< 70	1357 (50.5%)	1304 (96.1%)	53 (3.9%)		744 (56.0%)
≥ 70	1328 (49.5%)	1192 (89.8%)	136 (10.2%)		585 (44.0%)
LDH, U/L				< 0.001	
< 172.2	1344 (50.1%)	1287 (95.8%)	57 (4.2%)		706 (53.1%)
≥ 172.2	1341 (49.9%)	1209 (90.2%)	132 (9.8%)		623 (46.9%)
ALB, g/L				0.003	
< 44.9	1351 (50.3%)	1236 (91.5%)	115 (8.5%)		576 (43.3%)
≥ 44.9	1334 (49.7%)	1260 (94.5%)	74 (5.5%)		753 (56.7%)
GLB, g/L				0.507	
< 30.5	1341 (49.9%)	1251 (93.3%)	90 (6.7%)		793 (59.7%)
≥ 30.5	1344 (50.1%)	1245 (92.6%)	99 (7.4%)		536 (40.3%)
Cholesterol, mmol/L				0.054	
< 5.12	1353 (50.4%)	1245 (92.0%)	108 (8.0%)		576 (43.3%)
≥ 5.2	1332 (49.6%)	1251 (93.9%)	81 (6.1%)		753 (56.7%)
T lymphocytes, ×10^9^				0.289	
< 1.8	1392 (51.8%)	1287 (92.5%)	105 (7.5%)		622 (46.8%)
≥ 1.8	1293 (48.2%)	1209 (93.5%)	84 (6.5%)		707 (53.2%)
Monocytes, ×10^9^				0.005	
< 0.4	1385 (51.6%)	1306 (94.3%)	79 (5.7%)		462 (34.8%)
≥ 0.4	1300 (48.4%)	1190 (91.5%)	110 (8.5%)		867 (65.2%)
Pathology				0.852	
Undifferentiated	2592 (96.5%)	2410 (93.0%)	182 (7.0%)		1300 (97.8%)
Differentiated	93 (3.5%)	86 (92.5%)	7 (7.5%)		29 (2.2%)
Cranial nerve injury				0.730	
Absent	2498 (93.0%)	2321 (92.9%)	177 (7.1%)		1234 (92.9%)
Present	187 (7.0%)	175 (93.6%)	12 (6.4%)		95 (7.1%)
EBV-DNA, copies/ml				< 0.001	
< 1000	1130 (42.1%)	1092 (96.6%)	38 (3.4%)		526 (39.6%)
1000–9999	585 (21.8%)	555 (94.9%)	30 (5.1%)		265 (19.9%)
10,000–99,999	599 (22.3%)	555 (92.7%)	44 (23.3%)		325 (24.5%)
100,000–999,999	290 (10.8%)	245 (84.5%)	45 (15.5%)		156 (11.7%)
≥ 1,000,000	81 (3.0%)	49 (60.5%)	32 (39.5%)		57 (4.3%)
T category				0.804	
1	167 (6.2%)	158 (94.6%)	37 (5.4%)		81 (6.1%)
2	525 (19.6%)	488 (93.0%)	37 (7.0%)		328 (24.7%)
3	1374 (51.2%)	1278 (93.0%)	96 (7.0%)		630 (47.4%)
4	619 (23.1%)	572 (92.4%)	47 (7.6%)		290 (21.8%)
N category				< 0.001	
0	319 (11.9%)	312 (97.8%)	7 (2.2%)		250 (18.8%)
1	921 (34.3%)	887 (96.3%)	34 (3.7%)		449 (33.8%)
2	775 (28.9%)	697 (89.9%)	78 (10.1%)		370 (27.8%)
3	549 (20.4%)	494 (90.0%)	55 (10.0%)		243 (18.3%)
4	121 (4.5%)	106 (87.6%)	15 (12.4%)		17 (1.3%)
Radiotherapy technique				0.451	
IMRT +3DCRT	1341(49.9%)	1252 (93.4%)	89 (6.6%)		705(65.9%)
CRT	1344(51.1%)	1244 (92.6%)	100 (7.4%)		624(34.1%)
Treatment method				*P* < 0.001	
Radiotherapy	505(18.8%)	481 (95.2%)	24 (4.8%)		318 (24.1%)
CCRT	1136 (42.3%)	1086 (95.6%)	50(4.4%)		425 (32.2%)
Neo + radiotherapy	483 (18.0%)	419 (86.7%)	64 (13.3%)		265 (20.1%)
Neo + CCRT	561(20.9%)	510 (90.9%)	51(9.1%)		311 (23.5%)
SMAD					
Absent	2496 (93.0%)				1231 (92.6%)
Present	189 (7%)				98 (7.4%)

*Abbreviations: SMAD* skeletal metastasis at time of diagnosis, *WBCs* white blood cells, *HGB* hemoglobin, *GLB* globulin, *ALB* albumin, *ALT* alanine transaminase, *AST* aspartate transaminase, *ALP* alkaline phosphatase, *LDH* lactate dehydrogenase, *CRP* C-reactive protein, *GGT* gamma glutamyl transpeptidase, *EBV-DNA* Epstein-Barr virus DNA, *Undifferentiated* undifferentiated non-keratinizing carcinoma, *Differentiated* differentiated carcinoma, *CRT* conventional radiotherapy, *IMRT* intensity modulated radiation therapy, *3D–CRT* three dimensional conformal radiation therapy, *RT* radiotherapy, *CCRT* concurrent radiotherapy, *Neo* neoadjuvant chemotherapy

### Univariate and multivariate analyses

The factors associated with significantly poorer SMAD included in the univariate logistic regression model were sex (male); elevated LDH, CRP, ALP, platelets, monocytes, neutrophils and plasma EBV DNA; decreased hemoglobin (HGB) and ALB; and advanced clinical N category. All significant variables were entered into multivariate logistic regression; ALP, LDH, HGB, plasma EBV DNA and N category retained independent prognostic significance for SMAD.

The factors associated with significantly poorer SMFS in the univariate Cox regression models were advanced age; elevated LDH, CRP, ALP, monocytes and plasma EBV-DNA; decreased globulin (GLB) and ALB; and advanced clinical N category. ALP, LDH, CRP, plasma EBV DNA and N category retained independent prognostic value in multivariate logistic regression. Detailed summaries of the multivariate analyses are shown in Tables 2 and 3.

**Table 2:** Associations between the clinical and laboratory characteristics of the patients and SMAD in univariate and multivariate logistic regression analysis

Characteristic	Univariate			Multivariate		
	HR	95% CI	*P*-value	HR	95% CI	*P*-value
Age (≥ 45 vs. < 45 years)	1.142	0.850–1.535	0.379			
Gender (Male vs. Female)	0.623	0.410–0.946	0.027			
Smoking Status (Present vs. Absent)	1.139	0.993–1.807	0.056			
Drinking Status (Present vs. Absent)	1.038	0.655–1.647	0.873			
Family history (Present vs. Absent)	0.907	0.649–1.267	0.566			
Calcium, mmol/L (≥ 2.4 vs. < 2.4)	0.987	0.734–1.327	0.932			
Phosphorus, mmol/L (≥ 1.15 vs. < 1.15)	0.921	0.685–1.239	0.587			
Magnesium, mmol/L (≥ 0.93 vs. < 0.93)	0.857	0.636–1.154	0.308			
CRP, mg/L (≥ 1.91 vs. < 1.91)	2.167	1.583–2.965	< 0.001			
WBCs, ×10^9^ (≥ 6.9 vs. < 6.9)	1.252	0.931–1.684	0.137			
Neutrophils, ×10^9^ (≥ 4.2 vs. < 4.2)	1.681	1.241–2.276	0.001			
HGB, g/L (≥ 145 vs. < 145)	0.660	0.488–0.893	0.007	0.672	0.477–0.948	0.023
Platelets, ×10^9^ (≥ 229 vs. < 229)	1.462	1.083–1.974	0.013			
ALT, U/L (≥ 22.2 vs. < 22.2)	1.138	0.846–1.530	0.392			
AST, U/L (≥ 21 vs. < 21)	1.290	0.958–1.736	0.093			
ALP, U/L (≥ 70 vs. < 70)	2.807	2.024–3.893	< 0.001	2.148	1.509–3.056	< 0.001
LDH, U/L (≥ 172.2 vs. < 172.2)	2.465	1.789–3.396	< 0.001	1.512	1.069–2.139	0.019
ALB, g/L (≥ 44.9 vs. < 44.9)	0.631	0.466–0.854	0.003			
GLB, g/L (≥ 30.5 vs. < 30.5)	1.105	0.822–1.486	0.507			
Cholesterol, mmol/L (≥ 5.12 vs. < 5.12)	0.746	0.554–1.006	0.055			
T lymphocytes, ×10^9^ (≥ 1.8 vs. < 1.8)	0.852	0.632–1.147	0.290			
Monocytes, ×10^9^ (≥ 0.4 vs. < 0.4)	1.528	1.133–2.062	0.006			
Pathology (Differentiated vs. Undifferentiated	1.078	0.492–2.363	0.852			
Cranial nerve injury (Absent vs. Present)	0.899	0.491–1.646	0.899			
EBV-DNA, copies/ml			< 0.001			< 0.001
< 1000	1.000	1.000		1.000	1.000	
1000–9999	1.553	0.952–2.534	0.078	1.293	0.784–2.131	0.314
10,000–99,999	2.278	1.459–3.558	< 0.001	1.588	0.998–2.530	0.051
100,000–999,999	5.278	3.354–8.307	< 0.001	3.234	1.982–5.279	< 0.001
≥ 1,000,000	18.767	10.822–32.544	< 0.001	10.703	5.876–19.498	< 0.001
T category			0.805			
1	1.000	1.000				
2	1.331	0.629–2.818	0.455			
3	1.319	0.653–2.663	0.440			
4	1.443	0.692–3.007	0.328			
N category			< 0.001			0.002
0	1.000	1.000		1.000	1.000	
1	1.708	0.750–3.893	0.202	1.292	0.559–2.984	0.549
2	4.988	2.276–10.933	< 0.001	2.924	1.304–6.557	0.009
3	4.962	2.232–11.035	< 0.001	2.299	0.996–5.306	0.051
4	6.307	2.504–15.887	< 0.001	2.606	0.983–6.905	0.054

*Abbreviations: SMAD* skeletal metastasis at the time of diagnosis, *WBCs* white blood cells, *HGB* hemoglobin, *GLB* globulin, *ALB* albumin, *ALT* alanine transaminase, *AST* aspartate transaminase, *ALP* alkaline phosphatase, *LDH* lactate dehydrogenase, *CRP* C-reactive protein, *GGT* gamma glutamyl transpeptidase, *EBV-DNA* Epstein-Barr virus DNA, *Undifferentiated* undifferentiated non-keratinizing carcinoma, *Differentiated* differentiated carcinoma

**Table 3:** Associations between the clinical and laboratory characteristics of the patients and SMFS in univariate and multivariate logistic regression analysis

Characteristic	Univariate			Multivariate		
	HR	95% CI	*P*-value	HR	95% CI	*P*-value
Age (≥ 45 vs. < 45 years)	1.288	1.008–1.647	0.043			
Gender (Male vs. Female)	0.867	0.635–1.184	0.371			
Smoking Status (Present vs. Absent)	1.120	0.871–1.440	0.376			
Drinking Status (Present vs. Absent)	0.911	0.615–1.349	0.642			
Family history (Present vs. Absent)	0.831	0.627–1.010	0.198			
Calcium, mmol/L (≥ 2.4 vs. < 2.4)	0.927	0.725–1.186	0.548			
Phosphorus, mmol/L (≥ 1.15 vs. < 1.15)	0.927	0.725–1.185	0.545			
Magnesium, mmol/L (≥ 0.93 vs. < 0.93)	0.804	0.552–1.172	0.257			
CRP, mg/L (≥ 1.91 vs. < 1.91)	2.092	1.618–2.706	< 0.001	1.450	1.108–1.897	0.007
WBCs, ×10^9^ (≥ 6.9 vs. < 6.9)	1.050	0.822–1.342	0.694			
Neutrophils, ×10^9^ (≥ 4.2 vs. < 4.2)	1.177	0.921–1.504	0.193			
HGB, g/L (≥ 145 vs. < 145)	0.835	0.653–1.068	0.150			0.023
Platelets, ×10^9^ (≥ 229 vs. < 229)	1.134	0.887–1.449	0.315			
ALT, U/L (≥ 22.2 vs. < 22.2)	0.971	0.760–1.241	0.814			
AST, U/L (≥ 21 vs. < 21)	1.283	1.003–1.641	0.047			
ALP, U/L (≥ 70 vs. < 70)	2.023	1.570–2.606	< 0.001	1.654	1.275–2.145	< 0.001
LDH, U/L (≥ 172.2 vs. < 172.2)	1.951	1.514–2.514	< 0.001	1.424	1.098–1.847	< 0.001
ALB, g/L (≥ 44.9 vs. < 44.9)	0.694	0.542–0.889	0.004			
GLB, g/L (≥ 30.5 vs. < 30.5)	1.594	1.242–2.047	< 0.001			
Cholesterol, mmol/L (≥ 5.12 vs. < 5.12)	0.955	0.747–1.220	0.710			
T lymphocytes, ×10^9^ (≥ 1.8 vs. < 1.8)	0.913	0.714–1.167	0.468			
Monocytes, ×10^9^ (≥ 0.4 vs. < 0.4)	1.431	1.118–1.832	0.004			
Pathology (Differentiated vs. Undifferentiated	0.410	0.153–1.101	0.077			
Cranial nerve injury (Absent vs. Present)	1.075	0.666–1.736	0.767			
EBV-DNA, copies/ml			< 0.001			< 0.001
< 1000	1.000	1.000		1.000	1.000	
1000–9999	1.955	1.349–2.832	< 0.001	1.521	1.045–2.215	0.029
10,000–99,999	2.757	1.959–3.881	< 0.001	1.822	1.277–2.601	0.001
100,000–999,999	4.569	3.147–6.631	< 0.001	2.706	1.829–4.004	< 0.001
≥ 1,000,000	7.451	4.221–13.151	< 0.001	4.764	1.829–8.533	< 0.001
Treatment method				0.040		
Radiotherapy	1.000	1.000				
CCRT	1.064	0.639–1.773	0.811			
Neo + Radiotherapy	0.188	0.834–2.521	0.188			
Neo + CCRT	0.752	0.426–1.325	< 0.001			
Radiotherapy technology (IMRT + 3DCRT vs. CRT)	0.745	0.378–1.471	0.397			
T category			0.021			
1	1.000	1.000				
2	3.190	1.269–8.020	0.014			
3	3.752	1.538–9.157	0.004			
4	3.966	1.596–9.856	0.003			
N category			< 0.001			< 0.001
0	1.000	1.000		1.000	1.000	
1	1.731	0.928–3.230	0.085	1.432	0.765–2.681	0.262
2	3.017	1.638–5.558	< 0.001	2.149	1.156–3.995	0.016
3	5.987	3.281–10.925	< 0.001	3.613	1.947–6.704	< 0.001
4	6.310	3.079–12.933	< 0.001	3.629	1.742–7.559	0.001

*Abbreviations: SMFS* skeletal metastasis-free survival, *WBCs* white blood cells, *HGB* hemoglobin, *GLB* globulin, *ALB* albumin, *ALT* alanine transaminase, *AST* aspartate transaminase, *ALP* alkaline phosphatase, *LDH* lactate dehydrogenase, *CRP* C-reactive protein, *GGT* gamma glutamyl transpeptidase, *EBV-DNA* Epstein-Barr virus DNA, *Undifferentiated* undifferentiated non-keratinizing carcinoma, *Differentiated*, differentiated carcinoma

### Nomograms for predicting SMAD and SMFS

**Fig. 1:**
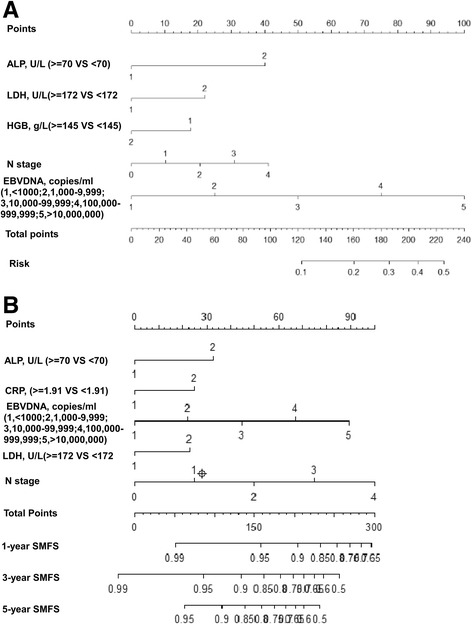
Nomograms for predicting SMAD (**a**) and SMFS (**b**) in NPC. Points refers to the value of each factor included in the nomogram; total points, total points for all factors; 1/3/5-year survival, survival probability based on total points; ALP, alkaline phosphatase; HGB, hemoglobin; LDH, lactate dehydrogenase; CRP, C-reactive protein; EBV, Epstein-Barr virus; SMAD, skeletal metastasis at diagnosis; SMFS, skeletal-metastasis free survival

The independent prognostic factors for SMAD and SMFS were used to construct nomograms (Fig. 1). Each variable was assigned a score. By determining the total score for all variables on the total point scale, the probabilities of specific outcomes could be determined by drawing a vertical line from the total score. Plasma EBV DNA copy number was the most important factor for prediction of both SMAD and SMFS.

In the training cohort, the SMAD nomogram had a bootstrap-corrected c-index of 0.83 (95% CI, 0.78–0.87), significantly higher than the TNM classification (0.73; 95% CI, 0.70–0.77; *P* = 0.005). The c-index of the nomogram for SMFS (0.70; 95% CI, 0.67–0.74) was also significantly higher than the TNM classification (0.59; 95% CI, 0.56–0.63; *P* < 0.001). In the external validation cohort, the c-index value of the nomogram for SMAD was 0.76 (95% CI, 0.71–0.79) and 0.61 (95% CI, 0.55–0.66) for SMFS; both of which were significantly better than the c-index values for the TNM classification with respect to SMAD (0.64; 95% CI, 0.60–0.67; *P* < 0.001) and SMFS (0.58; 95% CI, 0.54–0.63; *P* = 0.005), respectively (Table 4).

**Table 4:** The c-index values for performance of the multivariate model and the TNM classification for prediction of SMAD and SMFS in the training set and validation set

Model	Training set			Validation set		
	C-index	95% CI	*P-*value	C-index	95% CI	*P-*value
Nomograms (SMAD)	0.83	0.78–0.87	0.005	0.76	0.71–0.79	< 0.001
TNM classification (SMAD)	0.73	0.70–0.77		0.64	0.60–0.67	
Nomograms (SMFS)	0.70	0.67–0.74	< 0.001	0.61	0.55–0.0.66	0.005
TNM classification (SMFS)	0.59	0.56–0.63		0.58	0.54–0.63	

*Abbreviations: SMAD* skeletal metastasis at the time of diagnosis, *SMFS* skeletal metastasis-free survival

**Fig. 2:**
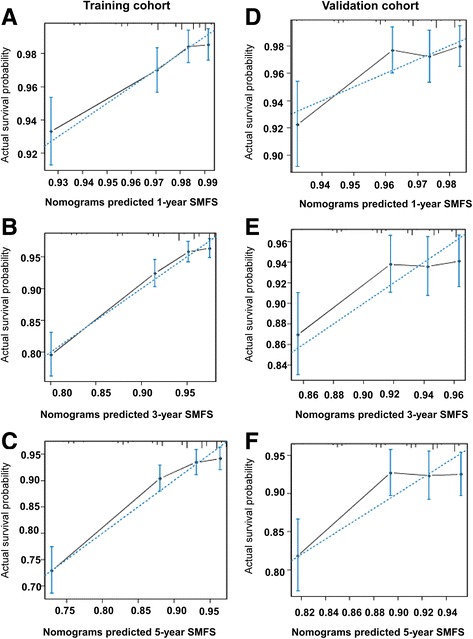
Calibration plots for SMFS at 1, 3 and 5 years in the training cohort (**a**, **b**, **c**) and validation cohort (**d**, **e**, **f**). Nomogram-predicted SMFS is plotted on the *x*-axis; actual rates of SMFS are plotted on the *y*-axis. The dashed lines along the 45-degree line through the origin represent the perfect calibration models in which the predicted probabilities are identical to the actual probabilities. SMFS, skeletal-metastasis free survival

The calibration plots demonstrated good agreement between the nomogram predictions and actual 1-, 3-, and 5-year SMFS rates observed in both the training and the validation cohorts (Fig. 2).

### Nomograms for risk stratification

**Fig. 3:**
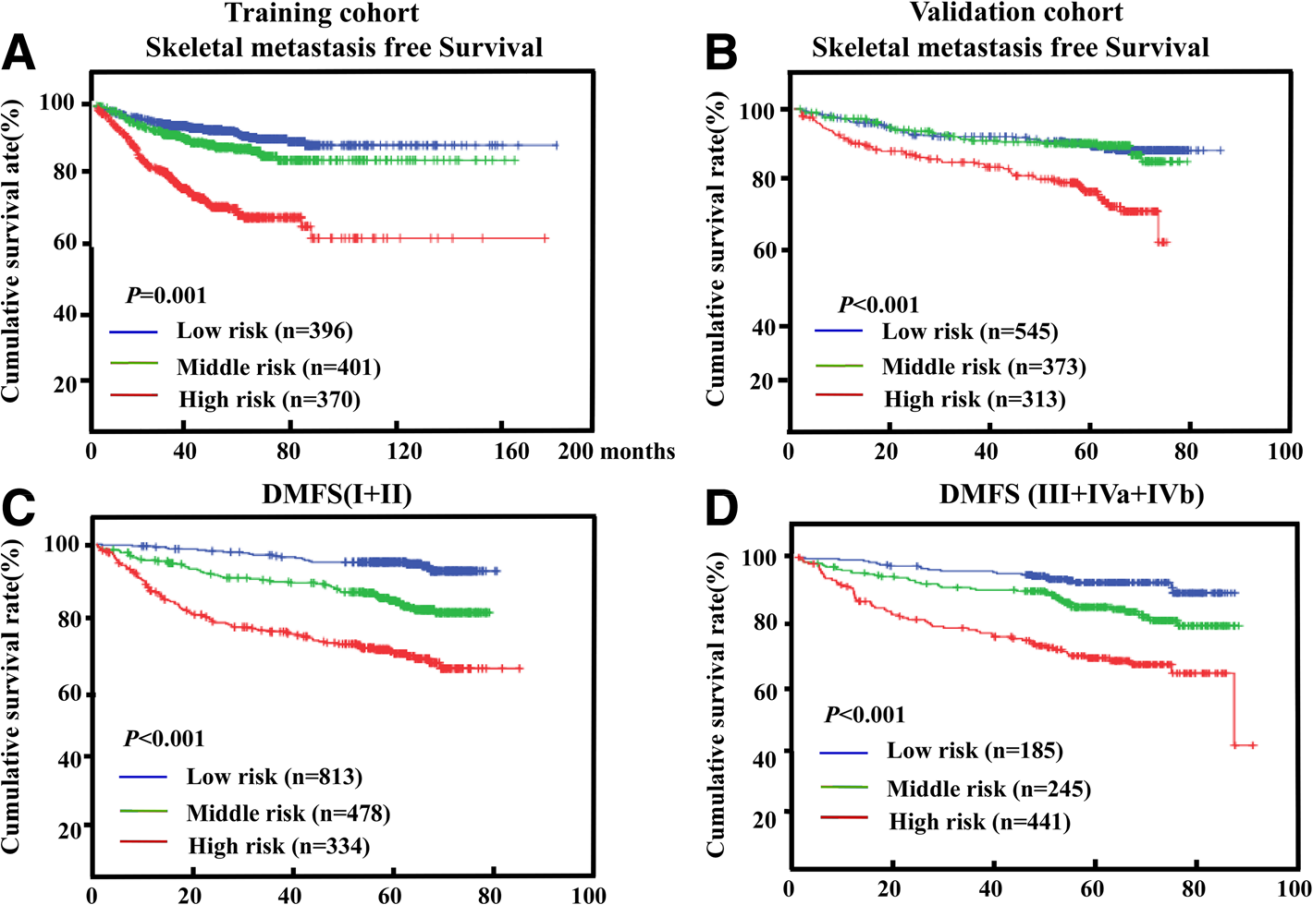
Kaplan–Meier curves of SMFS for the three risk group stratifications. Nomogram risk group stratifications for the 33 and 66 percentiles are shown for the training cohort (**a**, **c**) and validation cohort (**b**, **d**). SMFS, skeletal-metastasis free survival

We determined the cut-off values for the nomogram-generated scores by which the patients in the training cohort could be stratified into three risk groups. Each group had a distinct prognosis (Additional file 3: Table S2). This stratification could effectively predict SMFS for the three proposed risk groups in both the training and validation cohorts (Fig. 3). The risk stratification even provided significant distinction between the Kaplan-Meier SMFS curves for each of the three risk groups within each TNM stage (Fig. 3).

## Discussion

This is the first study to retrospectively assess a very large number of patients with NPC to evaluate the prognostic value of a wide range of clinical and laboratory parameters in order to establish effective prognostic tools for skeletal metastasis. The nomograms established in this analysis demonstrated superior discriminative ability compared to the TMM classification of the seventh edition of the UICC/AJCC staging system and enabled risk scoring for individual patients. The independent prognostic factors for skeletal metastasis (SMAD, SMFS) included N category, circulating EBV-DNA, LDH, ALP, HGB and CRP; each of these factors has been previously reported to play a vital role in tumor progression or metastasis.

Advanced N category was significantly associated with skeletal metastasis in this study, which reflects the assumption that the tumor cells responsible for distant metastasis disseminate from the lymph nodes, rather than the primary tumor. In agreement with our findings, high serum ALP has also previously been reported to be a negative prognostic factor for skeletal metastasis and is used in the clinic to predict the presence of bone metastases in a range of cancers, including lung cancer and prostate cancer [12, 13]. The hydrolase ALP dephosphorylates a variety of molecules. Serum ALP is usually low in healthy individuals, but increases during pregnancy and in patients with bile duct obstruction, kidney disease, hepatocellular carcinoma or bone metastasis [14–18]. Yang et al. reported a high serum LDH level was an independent, unfavorable risk factor for overall survival (OS) and distant-metastasis free survival (DMFS) in non-metastatic NPC [19]. This study provides the first evidence that high serum LDH is an independent prognostic factor for skeletal metastasis in NPC. Rapid tumor cell proliferation initiates anaerobic glycolysis to produce energy, which requires the transformation of pyruvate to lactate by LDH, a key enzyme of glycolysis [20]. In addition, increased LDH levels lead to a low extracellular pH and activate the hypoxia-inducible factor (HIF) pathway, which is well-recognized to promote tumor growth, aggressiveness and distant metastasis [21–25].

In the regions where NPC is endemic, EBV infection is associated with an increased risk of NPC, and plasma EBV DNA is a useful prognostic marker in both early and advanced NPC [26, 27]. The present study indicates that circulating EBV DNA is also an independent prognostic factor for skeletal metastasis in NPC. Leung et al. reported that the EBV DNA cutoff value of 4000 copies/mL could categorize patients with early-stage NPC into a high-risk subgroup (with similar survival outcomes to patients with stage III disease) and a low-risk subgroup (with similar survival outcomes to stage I disease) [28]. A previously-established nomogram for disease-free survival (DFS) revealed incorporation of plasma EBV DNA increased the C-index compared to the model that did not include EBV DNA [29]. In further confirmation of its prognostic value, plasma EBV DNA was incorporated as a significant factor into the prognostic models for SMAD and SMFS in this study, and resulted in more accurate risk discrimination for individual patients.

Reduced HGB was also an independent prognostic factor for poor SMAD, consistent with the report by Ong et al. [30]. Anemia is more common in patients with advanced stage disease and/or a poor performance status, both of which are associated with a higher probability of skeletal metastasis in NPC. Elevated CRP has been associated with advanced tumor classification, bone invasion and lymph node metastasis in NPC [31]. Similarly, CRP moderately enhanced the predictive ability of the SMFS nomogram in this study. The link between inflammation and cancer is well-recognized; prolonged exposure to proinflammatory cytokines may eventually result in the induction of CRP synthesis and is considered to be a prognostic factor in NPC [32, 33]. In the future, improving nutrition status, inflammatory status and immune function could potentially further improve the clinical outcome of patients with NPC.

The present study has several limitations. First, the time span of data collection was nearly 7 years for the data set. Therefore, the question of whether the nomograms can be applied to patients currently receiving treatment should be asked. However, at our institution, the pathologic examination has not changed during this period of time. Second, patient comorbidities were not assessed. Liu et al. previously reported that comorbidity could affect OS to some extent in NPC [34]. However, the diversity of comorbidities makes it difficult to establish categorized variables and quantify risk. Therefore, the prognostic significance of comorbidities should be assessed in future nomogram studies. Finally, whether this nomogram can be applied to younger patients (aged <18-years-old) or patients in areas with a low occurrence of NPC remains to be determined.

In summary, we have developed and externally-validated nomograms to predict SMAD and SMFS based on analyses of a relatively large number of patients with NPC. The nomograms provide significantly better discrimination than the current seventh TNM classification of the AJCC staging system and also enable individualized prognostication of skeletal metastasis. Moreover, the accuracy of the nomograms was validated using large datasets for patients treated at other two institutions. In conclusion, these nomograms represent useful tools for predicting skeletal metastasis, facilitating patient counseling, and providing timely surveillance and clinical assessments.

## Conclusion

This is the first large cohort study to establish a prediction nomogram for skeletal metastasis in non-metastatic NPC; the predictive accuracy of the model was validated in an external cohort.

## Additional files

Additional file1: R code of the nomograms for SMAD and SMFS in non-metastatic NPC after definitive radiotherapy. (DOCX 20 kb)

Additional file 2: Figure S1. Schematic of patient inclusion and exclusion. (TIFF 30204 kb)

Additional file 3: Table S1. Associations between clinical and laboratory characteristics and SMFS as indicated by the chi-square test or Fisher’s exact test. (DOC 174 kb)

## Abbreviations

18-FDG PET/CT: 18F–deoxyglucose positron emission tomography/computed tomography
ALB: Albumin
ALP: Alkaline phosphatase
ALT: Alanine transaminase
AST: Aspartate transaminase
AUC: Area under curve
BS: Bone scintigraphy
CRP: C-reactive protein
DMFS: Distant-metastasis free survival
EBV: Epstein-Barr virus
GLB: Globulin
HGB: Hemoglobin
HIF: Hypoxia-inducible factor
LDH: Lactate dehydrogenase
NPC: Nasopharyngeal carcinoma
OS: overall survival
RT: radiotherapy
SMAD: Skeletal metastasis at initial diagnosis
SMFS: Skeletal metastasis-free survival
