# Supplementary material for: Development and external validation of nomograms to predict the risk of skeletal metastasis at the time of diagnosis and skeletal metastasis-free survival in nasopharyngeal carcinoma
Source: BMC Cancer. 2017 Sep 6;17:628. doi: 10.1186/s12885-017-3630-9 (PMC5586019; doi:10.1186/s12885-017-3630-9)
Supplement: Supplementary file 1 — R code of the nomograms for SMAD and SMFS in non-metastatic NPC after definitive radiotherapy. (DOCX 20 kb) [file 12885_2017_3630_MOESM1_ESM.docx]

**Development and external validation of nomograms to predict the risk of skeletal metastasis at the time of diagnosis and skeletal metastasis-free survival in nasopharyngeal carcinoma**

**Short title**: Nomograms for skeletal metastasis in NPC

Lin. Yang^1,2,3#^, Liangping. Xia^1,2,3#^, Yan. Wang^1,2,3#^, Shasha. He^1,2,3,^Haiyang. Chen^4^, Shaobo. Liang^5^, Peijian. Peng^6^, Shaodong Hong^1,2,3*^_,_Yong. Chen^1, 2,3^*

**R code Nomogram for the SMAD and SMFS non-metastatic NPC after definitive radiotherapy**

This is the R code of nomogram development and validation of the primary cohort using R software. (The validation was conducted by the biostatisticians using the same methods.)

**#construction of nomograms**

setwd("D:/")

library(MASS)

library(foreign)

library(splines)

library(rms)

lc<-read.spss("NPC_training.sav",use.value.labels=T,to.data.frame=T)

attach(lc)

coxm<-cph(Surv(OS,OSstatus)~age+gender+LDH+CRP+Tstage+Nstage+EBV,x=T,y=T,data=lc,surv=T)

scoxm<-step(coxm)

dd<-datadist(lc)

options(datadist="dd")

surv<-Survival(scoxm)

surv1<-function(x) surv(1*365,lp=x)

surv2<-function(x) surv(3*365,lp=x)

surv3<-function(x) surv(5*365,lp=x)

nom <-nomogram(scoxm,fun=list(surv1,surv2,surv3),lp=F,funlabel=c('1-year survival','3-year survival','5-year survival'),maxscale=100,fun.at=c (1.00,0.95,0.9,0.85,0.8,0.75,0.7,0.6,0.5,0.4,0.3,0.2,0.1,0))

plot(nom, xfrac=.45)

print(nom)

**#resampling internal validation**

set.seed(1)

validate(coxm, B=1000, dxy=TRUE)

**#calibration @1 years OS**

coxm<-cph(Surv(OS,OSstatus)~age+gender+LDH+CRP+Tstage+Nstage+EBV,x=T,y=T,data=lc,surv=T,time.inc=365)

cal<-calibrate(coxm, cmethod='KM', method='boot',u=365,B=10)

cal

plot(cal, errbar.col = c(rgb(0, 255, 0, maxColorValue = 255)),col = c(rgb(0, 124, 194, maxColorValue = 255)))

box(lwd = 2)

abline(0, 1, lty =4, lwd = 2, col = c(rgb(0, 0, 0, maxColorValue = 255)))

**# calibration @3 years OS**

coxm<-cph(Surv(OS,OSstatus)~age+gender+LDH+CRP+Tstage+Nstage+EBV,x=T,y=T,data=lc,surv=T,time.inc=1095)

cal<-calibrate(coxm, cmethod='KM', method='boot',u=1095,B=10)

cal

plot(cal, errbar.col = c(rgb(0, 255, 0, maxColorValue = 255)),col = c(rgb(0, 124, 194, maxColorValue = 255)))

box(lwd = 2)

abline(0, 1, lty =4, lwd = 2, col = c(rgb(0, 0, 0, maxColorValue = 255)))

**# calibration @5 years OS**

coxm<-cph(Surv(OS,OSstatus)~age+gender+LDH+CRP+Tstage+Nstage+EBV,x=T,y=T,data=lc,surv=T,time.inc=1825)

cal<-calibrate(coxm, cmethod='KM', method='boot',u=1825,B=10)

cal

plot(cal, errbar.col = c(rgb(0, 255, 0, maxColorValue = 255)),col = c(rgb(0, 124, 194, maxColorValue = 255)))

box(lwd = 2)

abline(0, 1, lty =4, lwd = 2, col = c(rgb(0, 0, 0, maxColorValue = 255)))

**#calculation of c-index**

f<-predict(coxm)

x=rcorr.cens(f,Surv(OS,OSstatus))

se<- x["S.D."]/2

Low95 <- 1-x["C Index"] - 1.96*se

Upper95 <- 1-x["C Index"] + 1.96*se

cbind(1-x["C Index"], Low95, Upper95)

**#calculation of c-index for TNM staging**

x3<- rcorr.cens(stage,Surv(OS,OSstatus))

se<- x3["S.D."]/2

Low95 <- 1-x3["C Index"] - 1.96*se

Upper95 <- 1-x3["C Index"] + 1.96*se

cbind(1-x3["C Index"], Low95, Upper95)

**#comparison of c-index between different models**

rcorrp.cens(x1, x2, S, Surv(OS, Status))
