# Supplementary figures and images for: Development and external validation of nomograms to predict the risk of skeletal metastasis at the time of diagnosis and skeletal metastasis-free survival in nasopharyngeal carcinoma
Source: BMC Cancer. 2017 Sep 6;17:628. doi: 10.1186/s12885-017-3630-9 (PMC5586019; doi:10.1186/s12885-017-3630-9)

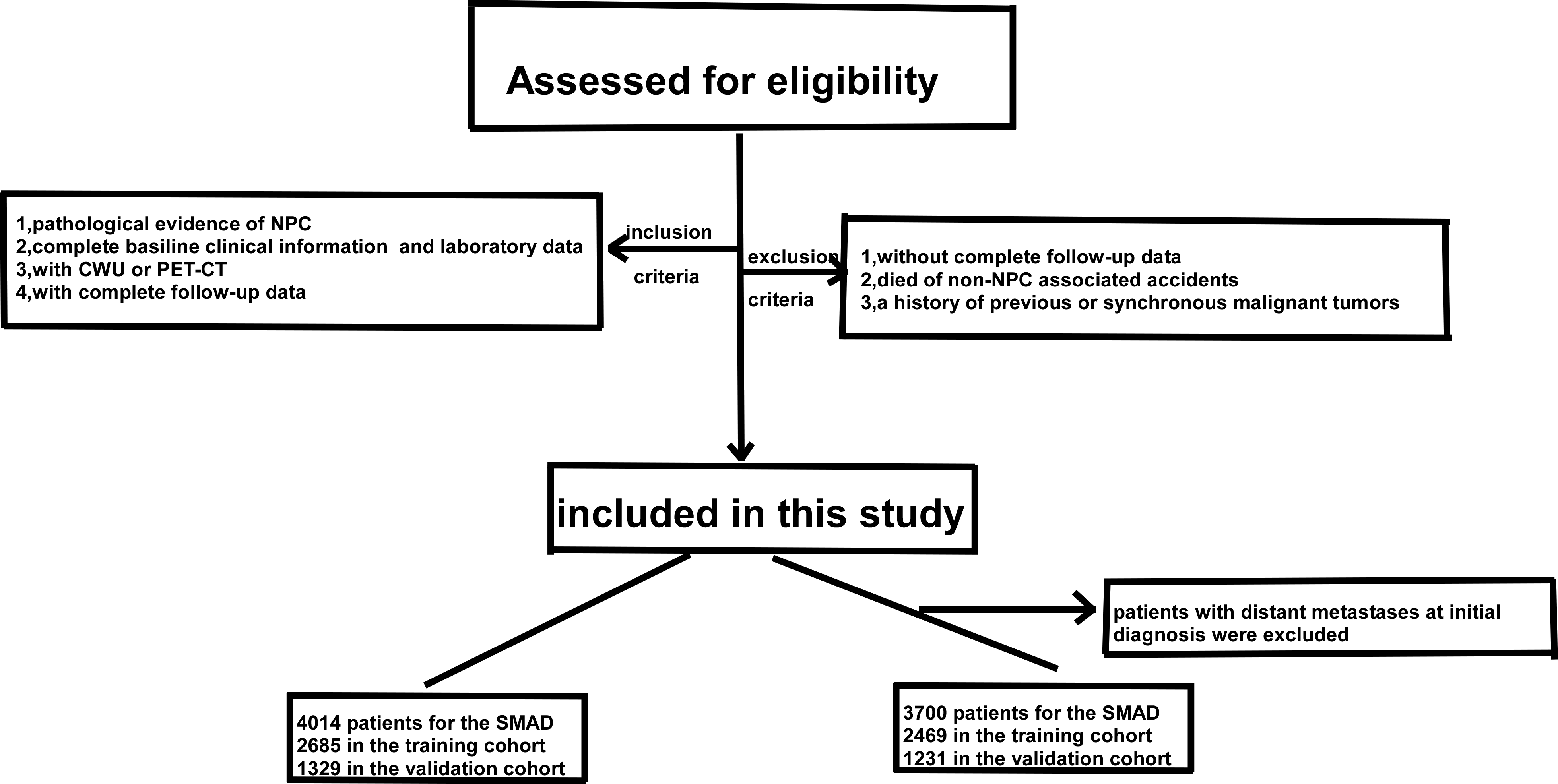

Supplement: Supplementary file 2 — Schematic of patient inclusion and exclusion. (TIFF 30204 kb) [file 12885_2017_3630_MOESM2_ESM.tif]
