# Supplementary material for: Development and external validation of nomograms to predict the risk of skeletal metastasis at the time of diagnosis and skeletal metastasis-free survival in nasopharyngeal carcinoma
Source: BMC Cancer. 2017 Sep 6;17:628. doi: 10.1186/s12885-017-3630-9 (PMC5586019; doi:10.1186/s12885-017-3630-9)
Supplement: Supplementary file 3 — Associations between clinical and laboratory characteristics and SMFS as indicated by the chi-square test or Fisher’s exact test. (DOC 174 kb) [file 12885_2017_3630_MOESM3_ESM.doc]

**Additional file 3**

**Table S1 Associations between the clinical and laboratory characteristics of the patients and SMFS as indicated by the chi-square test or Fisher’s exact test**

| **Characteristic** | **Number (%)** | **Training cohort** | | ***P*-value** | **Validation cohort**  **Number (%)** |
| --- | --- | --- | --- | --- | --- |
|  |  | **SMFS** | |  |  |
|  |  | **Absent** | **Present** |  |  |
| **Age, years** |  |  |  | 0.100 |  |
| < 45 | 1311 (52.5%) | 1189 (90.7%) | 122 (9.3%) |  | 629 (51.1%) |
| ≥ 45 | 1185 (47.5%) | 1051 (88.7%) | 134 (11.3%) |  | 602 (48.9%) |
| **Gender** |  |  |  | 0.414 |  |
| Male | 1969 (78.9%) | 1762 (89.5%) | 207 (10.5%) |  | 902 (73.3%) |
| Female | 527 (21.1%) | 478 (90.7%) | 49 (9.3%) |  | 329 (26.7%) |
| **Smoking status** |  |  |  | 0.265 |  |
| Absent | 1600 (64.1%) | 1444 (90.3%) | 156 (9.8%) |  | 739 (60.0%) |
| Present | 896 (35.9%) | 796 (88.8%) | 100 (11.2%) |  | 492 (40.0%) |
| **Drinking status** |  |  |  | 0.864 |  |
| Absent | 2215 (88.7%) | 1987 (89.7%) | 228 (10.3%) |  | 1029 (83.6%) |
| Present | 281 (11.3%) | 253 (90.0%) | 28 (10.0%) |  | 202 (16.4%) |
| **Family history** |  |  |  | 0.259 |  |
| Absent | 1787 (71.6%) | 1596 (89.3%) | 191 (10.7%) |  | 892 (72.5%) |
| Present | 709 (28.4%) | 644 (90.8%) | 65 (9.2%) |  | 339 (27.5%) |
| **Calcium, mmol/L** |  |  |  | 0.638 |  |
| < 2.4 | 1273 (51.0%) | 1146 (90.0%) | 127 (10.0%) |  | 444 (36.1%) |
| ≥ 2.4 | 1223 (49.0%) | 1094 (89.5%) | 129 (10.5%) |  | 787 (63.9%) |
| **Phosphorus, mmol/L** |  |  |  | 0.685 |  |
| < 1.15 | 1296 (51.9%) | 1160 (89.5%) | 136 (10.5%) |  | 623 (50.6%) |
| ≥ 1.15 | 1200 (48.1%) | 1080 (90.0%) | 120 (10.0%) |  | 608 (49.4%) |
| **Magnesium, mmol/L** |  |  |  | 0.023 |  |
| < 0.93 | 1304 (52.2%) | 1153 (88.4%) | 151 (11.6%) |  | 869 (70.6%) |
| ≥ 0.93 | 1192 (47.8%) | 1087 (91.2%) | 105 (8.8%) |  | 362 (29.4%) |
| **CRP, mg/L** |  |  |  | < 0.001 |  |
| < 1.91 | 1283 (51.4%) | 1194 (93.1%) | 89 (6.9%) |  | 696 (56.5%) |
| ≥ 1.91 | 1213 (48.6%) | 1046 (86.2%) | 167 (13.8%) |  | 535 (43.5%) |
| **WBCs, ×10^9^** |  |  |  | 0.771 |  |
| < 6.9 | 1289 (51.6%) | 1159 (89.9%) | 130 (10.1%) |  | 659 (53.5%) |
| ≥ 6.9 | 1207 (48.4%) | 1081 (89.6%) | 126 (10.4%) |  | 572 (46.5%) |
| **Neutrophils, ×10^9^** |  |  |  | 0.257 |  |
| < 4.2 | 1283 (51.4%) | 1160 (90.4%) | 123 (9.6%) |  | 659 (53.5%) |
| ≥ 4.2 | 1213 (48.6%) | 1080 (89.0%) | 133 (11.0%) |  | 572 (46.5%) |
| **HGB, g/L** |  |  |  | 0.217 |  |
| < 145 | 1264 (50.6%) | 1125 (89.0%) | 139 (11.0%) |  | 699 (56.8%) |
| ≥ 145 | 1232 (49.4%) | 1115 (90.5%) | 117 (9.5%) |  | 532 (43.2%) |
| **Platelets, ×10^9^** |  |  |  | 0.307 |  |
| < 229 | 1265 (50.7%) | 1143 (90.4%) | 122 (9.6%) |  | 594 (48.3%) |
| ≥ 229 | 1231 (49.3%) | 1097 (89.1%) | 134 (10.9%) |  | 637 (51.7%) |
| **ALT, U/L** |  |  |  | 0.774 |  |
| < 22.2 | 1256 (50.3%) | 1125 (89.6%) | 131 (10.4%) |  | 685 (55.6%) |
| ≥ 22.2 | 1240 (49.7%) | 1115 (89.9%) | 125 (10.1%) |  | 546 (44.4%) |
| **AST, U/L** |  |  |  | 0.058 |  |
| < 21 | 1281 (51.3%) | 1164 (90.9%) | 117 (9.1%) |  | 632 (51.3%) |
| ≥ 21 | 1215 (48.7%) | 1076 (88.6%) | 139 (11.4%) |  | 599 (48.7%) |
| **ALP, U/L** |  |  |  | < 0.001 |  |
| < 70 | 1304 (52.2%) | 1208 (92.6%) | 96 (7.4%) |  | 723 (58.7%) |
| ≥ 70 | 1192 (47.8%) | 1032 (86.6%) | 160 (13.4%) |  | 508 (41.3%) |
| **LDH, U/L** |  |  |  | < 0.001 |  |
| < 172.2 | 1287 (51.6%) | 1191 (92.5%) | 96 (7.5%) |  | 680 (55.2%) |
| ≥ 172.2 | 1209 (48.4%) | 1049 (86.8%) | 160 (13.2%) |  | 551 (44.8%) |
| **ALB, g/L** |  |  |  | 0.016 |  |
| < 44.9 | 1236 (49.5%) | 1091 (88.3%) | 145 (11.7%) |  | 516 (41.9%) |
| ≥ 44.9 | 1260 (50.5%) | 1149 (91.2%) | 111 (8.8%) |  | 715 (58.1%) |
| **GLB, g/L** |  |  |  | 0.001 |  |
| < 30.5 | 1251 (50.1%) | 1147 (91.7%) | 104 (8.3%) |  | 755 (61.3%) |
| ≥ 30.5 | 1245 (49.9%) | 1093 (87.8%) | 152 (12.2%) |  | 476 (38.7%) |
| **Cholesterol, mmol/L** |  |  |  | 0.863 |  |
| < 5.12 | 1245 (49.49%) | 1116 (89.6%) | 129 (10.4%) |  | 566 (46%) |
| ≥ 5.2 | 1251 (50.1%) | 1124 (89.8%) | 127 (10.2%) |  | 665 (54%) |
| **T lymphocytes, ×10^9^** |  |  |  | 0.509 |  |
| < 1.8 | 1287 (51.6%) | 1150 (89.4%) | 137 (10.6%) |  | 568 (46.1%) |
| ≥ 1.8 | 1209 (48.4%) | 1090 (90.2%) | 119 (9.8%) |  | 663 (53.9%) |
| **Monocytes, ×10^9^** |  |  |  | 0.004 |  |
| < 0.4 | 1306 (52.3%) | 1194 (91.4%) | 112 (8.6%) |  | 425 (34.5%) |
| ≥ 0.4 | 1190 (47.7%) | 1046 (87.9%) | 144 (12.1%) |  | 806 (65.5%) |
| **Pathology** |  |  |  | 0.101 |  |
| Undifferentiated | 2410 (96.6%) | 2158 (89.5%) | 252 (10.5%) |  | 1203 (97.7%) |
| Differentiated | 86 (3.4%) | 82 (95.3%) | 4 (4.7%) |  | 28 (2.3%) |
| **Cranial nerve injury** |  |  |  | 0.989 |  |
| Absent | 2321 (93.0%) | 2083 (89.7%) | 238 (10.3%) |  | 1146 (93.1%) |
| Present | 175 (7.0%) | 157 (89.7%) | 18 (10.3%) |  | 85 (6.9%) |
| **EBV-DNA, copies/ml** |  |  |  | < 0.001 |  |
| < 1000 | 1092 (43.8%) | 1034 (94.7%) | 58 (5.3%) |  | 513 (41.7%) |
| 1000-9999 | 555 (22.2%) | 501 (90.3%) | 54 (9.7%) |  | 243 (19.7%) |
| 10,000-99,999 | 555 (22.2) | 479 (86.3%) | 76 (13.7%) |  | 302 (24.5%) |
| 100,000-999,999 | 245 (9.8%) | 192 (78.4%) | 53 (21.6%) |  | 133 (10.8%) |
| ≥ 1,000,000 | 49 (2.0%) | 34 (69.4%) | 15 (30.6%) |  | 40 (3.2%) |
| **Treatment method** |  |  |  | 0.103 |  |
| Radiotherapy | 469 (18.8%) | 364 (77.7%) | 105 (22.3%) |  | 297 (24.1%) |
| CCRT | 1056 (42.3%) | 818 (77.5%) | 238 (22.5%) |  | 396 (32.2%) |
| Neo-Radiotherapy | 449 (18.0%) | 119 (75.2%) | 330 (24.8%) |  | 247 (20.1%) |
| Neo+CCRT | 522 (20.9%) | 364 (69.7%) | 158 (30.3%) |  | 289 (23.5%) |
| **Radiotherapy** |  |  |  | 0.933 |  |
| IMRT+3DCRT | 1061 (52.5%) | 800 (75.4%) | 261 (24.6%) |  | 811 (65.9%) |
| CRT | 1435 (57.5%) | 1085 (75.6%) | 350 (24.2%) |  | 418 (34.1%) |
| **T category** |  |  |  | 0.018 |  |
| 1 | 158 (6.3%) | 153 (96.8%) | 5 (3.2%) |  | 78 (6.3%) |
| 2 | 488 (19.6%) | 441 (90.4%) | 47 (9.6%) |  | 312 (25.3%) |
| 3 | 1278 (51.2%) | 1138 (89.0%) | 140 (11.0%) |  | 581 (47.2%) |
| 4 | 572 (22.9%) | 508 (88.8%) | 64 (11.2%) |  | 260 (21.1%) |
| **N category** |  |  |  |  |  |
| 0 | 312 (12.5%) | 300 (96.2%) | 12 (3.8%) |  | 247 (20.1%) |
| 1 | 887 (35.5%) | 831 (93.7%) | 56 (6.3%) |  | 435 (35.3%) |
| 2 | 697 (27.9%) | 624 (89.5%) | 73 (10.5%) |  | 331 (26.9%) |
| 3 | 494 (19.8%) | 399 (80.8%) | 95 (19.2%) |  | 211 (17.1% |
| 4 | 106 (4.2%) | 86 (81.1%) | 20 (18.9%) |  | 7 (6%) |
| **Skeletal metastasis** |  |  |  |  |  |
| Present | 256 (10.3%) |  |  |  | 135 (11.0%) |
| Absent | 2240 (89.7%) |  |  |  | 1096 (89.0%) |

Abbreviations: SMFS, skeletal metastasis-free survival; WBCs, white blood cells; HGB, hemoglobin; GLB, globulin; ALB, albumin; ALT, [alanine transaminase; AST, aspartate transaminase; ALP, alkaline phosphatase; LDH, lactate dehydrogenase; CRP, C-reactive protein; GGT, gamma glutamyl transpeptidase; EBV-DNA, Epstein-Barr virus DNA;](http://dict.cnki.net/dict_result.aspx?searchword=谷丙转氨酶(alt)&tjType=sentence&style=&t=alanine+transaminase+(alt)) Undifferentiated, undifferentiated non-keratinizing carcinoma; Differentiated, differentiated carcinoma; CRT, conventional radiotherapy: IMRT, intensity-modulated radiation therapy; 3D-CRT, three dimensional conformal radiation therapy; RT, radiotherapy; CCRT, concurrent radiotherapy; Neo, neoadjuvant chemotherapy.

**Table S2 Point assignments from the** nomograms and prognostic scores

| **Characteristic** | **SMAD** | | **SMFS** | |
| --- | --- | --- | --- | --- |
|  | **Score** | **Estimated 5-year OS** | **Score** | **Estimated 5-year SMFS** |
| **HGB, g/L** |  |  |  |  |
| < 145 |  |  |  |  |
| ≥ 145` |  |  |  |  |
| **ALP, U/L** |  |  |  |  |
| < 70 |  |  | 0 |  |
| ≥ 70 |  |  | 22 |  |
| **LDH, U/L** |  |  |  |  |
| < 166 |  |  | 0 |  |
| ≥ 166 |  |  | 22 |  |
| **CRP, mg/L** |  |  |  |  |
| < 1.49 |  |  | 0 |  |
| ≥ 1.49 |  |  | 25 |  |
| **EBV-DNA, copies/ml** |  |  |  |  |
| < 1,000 |  |  | 0 |  |
| 1,000-9,999 |  |  | 22 |  |
| 10,000-99,999 |  |  | 45 |  |
| 100,000-999,999 |  |  | 67 |  |
| ≥ 1,000,0000 |  |  | 89 |  |
| **N category** |  |  |  |  |
| 0 |  |  | 0 |  |
| 1 |  |  | 25 |  |
| 2 |  |  | 50 |  |
| 3 |  |  | 75 |  |
| 4 |  |  | 100 |  |
| **Total prognostic score** |  |  |  |  |
| **Training cohort** |  |  |  |  |
| Low risk | < 72 | 96.2 | < 72 | 95.3 |
| Medium risk | 72-119 | 87.7 | 72-119 | 87.4 |
| High risk | >119 | 66.8 | >119 | 73.0 |
| **Validation cohort** |  |  |  |  |
| Low risk | < 72 | 94.3 | < 72 | 92.4 |
| Medium risk | 72-119 | 84.4 | 72-119 | 85.0 |
| High risk | >119 | 69.5 | >119 | 69.8 |

Abbreviations: HGB, hemoglobin; ALP, alkaline phosphatase; [LDH, lactate dehydrogenase; CRP, C-reactive protein; EBV-DNA, Epstein-Barr virus DNA;](http://dict.cnki.net/dict_result.aspx?searchword=谷丙转氨酶(alt)&tjType=sentence&style=&t=alanine+transaminase+(alt)) SMAD, skeletal metastasis at time of diagnosis; SMFS, skeletal metastasis-free survival.
